# Functional and Mechanistic Studies of the *EST* Gene in the Gonads of *Chlamys farreri*

**DOI:** 10.3390/biology15181574

**Published:** 2026-09-08

**Authors:** Yuxin Li, Wenzhu Cui, Xiaoling Liu

**Affiliations:** College of life Science, Yantai University, Yantai 264006, China; liyvxin2000@gmail.com (Y.L.); 14794575588@163.com (W.C.)

**Keywords:** *EST*, RNAi, ELISA, prokaryotic expression, enzyme activity assay, histological analysis

## Abstract

Estrogen sulfotransferase (EST), a member of the sulfotransferase (SULT) family, catalyzes estrogen sulfation and regulates its activity and content. EST has been mainly studied in vertebrates. Our previous analysis of EST expression in kidney, hepatopancreas, mantle, adductor muscle, gill, and gonads of *Chlamys farreri* (*C. farreri*) revealed highest expression in the gonads, suggesting an important role in gonadal function. We knocked down EST by RNAi, examined gonadal histology and E2 levels (via ELISA), expressed recombinant EST in prokaryotes, and assessed its activity using a novel MCF-7 proliferation assay, where E2 promotes cell growth and EST inactivation reduces it. After EST knockdown, EST mRNA levels decreased by 67% in the ovary and 63% in the testis, indicating effective silencing. Histological analysis revealed that RNAi accelerated oocyte proliferation and development, enhanced oocyte vitellogenesis and delayed testicular development in the experimental group. Moreover, estradiol (E2) levels in both ovaries and testes were higher in the experimental groups than in the controls (*p* < 0.05). The recombinant EST protein was expressed as inclusion bodies and, after purification, denaturation, refolding, and ultrafiltration, yielded high-purity, concentrated protein. Enzyme activity assays showed a decreased proliferation rate of MCF-7 cells, indicating that EST possesses enzymatic activity that can inactivate E2. It is worth mentioning that the traditional isotope-labeling method for detecting EST activity is limited by high cost, operational complexity, technical demands, and potential health risks; therefore, this study established a simpler, more economical, and safer method for measuring EST enzymatic activity. In conclusion EST contributes to the maintenance of estrogen balance during gonadal development.

## Simple Summary

This study addresses the high cost and safety risks of traditional isotope methods for detecting EST activity, aiming to clarify EST’s function in *Chlamys farreri* gonadal development and establish a novel assay. RNAi knockdown accelerated oocyte proliferation and development, delayed testicular development, and elevated E2 levels. Additionally, a safer, more economical enzyme activity assay was successfully developed based on MCF-7 cell proliferation using purified recombinant EST. In conclusion, EST regulates estrogen balance during scallop gonadal development. Scientifically, this advances knowledge of marine invertebrate reproduction; socially and economically, it provides a green, cost-effective, and safe technical alternative for enzymatic research.

## 1. Introduction

At present, research on estrogen sulfotransferase (*EST*) has focused almost exclusively on higher mammals. In humans (*Homo sapiens*), *EST* was also known as *SULT1E1*, it encodes estrogen sulfotransferase and belongs to the sulfotransferase (*SULT*) gene family. The SULT family comprises enzymes which can catalyze sulfation metabolism in the body; they catalyze the transfer of a sulfate group from the universal sulfate donor PAPS (3′-phosphoadenosine-5′-phosphosulfate) to hydroxyl or amino residues on various substrates. The above reaction was named sulfation [1]. In vertebrates, sulfation is generally regarded as a detoxification pathway. It includes two steps: First exogenous substances and small endogenous molecules can be sulfonated into water-soluble products, which are then excreted from the kidneys or bile [1,2]. Excessive estrogens are harmful to the body; for example, absolute estrogen concentrations were elevated in female mice. *Mus musculus* can cause placental thrombosis and spontaneous fetal loss [3], and this toxicity can be eliminated through estrogen sulfation. Within the *SULT* subfamily of humans and mice, *SULT1E1* specially acts on steroid hormones, such as estrone (E1) and estradiol (E2), and shows its highest catalytic efficiency during the sulfation of these substrates. Accordingly, *SULT1E1* is commonly referred to as estrogen sulfotransferase (*EST*) [4,5].

It is still uncertain whether steroid hormones are synthesized endogenously or obtained exogenously, but the presence of sex steroid hormones, such as testosterone, estradiol, and progesterone, has been extensively documented in mollusks. As early as the beginning of the 20th century, researchers identified traces of these hormones within the classes Cephalopoda, Gastropoda, and Bivalvia [6,7]. Subsequently, advancements in mass spectrometry and immunological techniques—such as gas chromatography–mass spectrometry (GC-MS), liquid chromatography–mass spectrometry (LC-MS), radioimmunoassay (RIA), and enzyme-linked immunosorbent assay (ELISA)—have provided direct and precise confirmation of various sex steroid hormones, including progesterone, testosterone, and estradiol, in bivalve species such as the blue mussel (*Mytilus edulis*) and the soft-shell clam (*Mya arenaria*) [8,9,10]. Liu et al. [11] quantified E2 levels and oocyte diameters in *C. farreri* ovaries at different gonad development stages; the researchers believed that ovarian E2 content is positively correlated with oocyte diameter, and E2 plays a crucial role in promoting oocyte growth and ovarian development. They observed a correlation between steroid hormone levels and activity and gonad development in *Chlamys farreri*.

Current studies on *EST* sequence characterization and tissue expression have mainly focused on humans [11], mice [12], and cattle (*Bovine*) [13], with *EST* expression mainly detected in the gonads and liver. In humans and mice, research of *EST* has further delved into functional and mechanistic investigations. For example, Gershon et al. [14] observed placental thrombosis and impaired ovulation by knocking out the *EST* gene in female mice, which ultimately led to reduced fertility. Based on these findings, the authors proposed that *EST* is a critical modulating factor of estrogen activity in the placenta. Further, Negishi et al. [15] elucidated the molecular mechanism of human *EST* and found that the EST protein catalyzes the transfer of a sulfate group from the donor PAPS to the hydroxyl group of E2, thereby promoting E2 sulfation. This modification prevents E2 from binding to its receptor, converting biologically active E2 into its inactive sulfated form.

It is worth mentioning that investigations of EST enzymatic activity have so far been restricted to humans, with all reported assays relying on radioisotope labeling methods [16,17,18]. For example, Rubin et al. [16] expressed recombinant human EST in *Escherichia coli* and employed 35S-labeled PAPS together with 3H-labeled estrogens as substrates. Their results demonstrated that human EST utilizes PAPS as the sulfate donor to catalyze sulfation at the 3′-hydroxyl group of estrogens, thereby rendering them inactive. However, radioisotope labeling approaches are associated with several limitations, including high cost, experimental complexity, high technical demands, and potential health risks. These constraints may partly explain the absence of EST enzymatic studies in mollusks. Consequently, there is an urgent need to establish more convenient, cost-effective, and safe methods for assessing EST enzymatic activity.

*Chlamys farreri* are the third largest marine cultured economic shellfish in China, with a production of 1.947 million tons in 2024, second only to oysters (7.252 million tons) and clams (4.738 million tons) [19]. Previous studies analyzed the amino acid sequence of *EST* in *Chlamys farreri* [20,21]. The results showed that *cf*-*EST* contains conserved dimerization regions, PAPS-binding domains, and amino acid residues (Ser, Lys, and His). However, two conserved amino acids forming the “gate” structure of the substrate-binding pocket differ from those observed in vertebrate *EST* genes. In addition, tissue (including the kidney, hepatopancreas, mantle, adductor muscle, gill, testis and ovary) expression analysis suggested that *cf*-*EST* plays an important function in gonadal development, although its precise biological function and regulatory mechanism remain unclear. Based on these preliminary findings, the present study further investigates the specific functions and underlying mechanisms of *cf*-*EST*. This work not only provides an experimental foundation for elucidating the role of *EST* in scallop gonadal development but also offers new insights for practical scallop aquaculture and seed production.

## 2. Materials and Methods

### 2.1. Animal Samples

Scallops (*C. farreri*) were obtained from a scallop aquaculture farm located in the Fushan District (Yantai, China). Healthy individuals at the growth stage were temporarily held in aerated (we first determined the growth stage through tissue sectioning and gonad color [22], and then selected scallops at the same developmental stage as experimental animals based on shell height and gonad color in order to ensure synchronous growth and development of experimental individuals (two-year-old scallop)), filtered seawater at approximately 16 °C (between 15.5 °C and 16.5 °C), with seawater replaced daily. Temperature was monitored daily, and all groups were maintained under identical conditions. During the holding period, the scallops were fed a mixed diet of diatoms and green algae. After 3 days of holding in the laboratory, subsequent experiments were conducted. The shell height of the experimental scallops averaged 67.11 ± 3.55 mm. Only scallops with intact shells, no visible external lesions, a rapid shell-closing response upon mild stimulation, and normal behavioral responses (including mantle retraction when stimulated) were used. Shell-closing performance has been shown to reflect the physiological condition of *C. farreri* [23].

### 2.2. dsRNA Design and Synthesis

Based on the *C. farreri EST* gene sequence (GenBank: PX515006), primers E3 and E4 were designed and synthesized (Table 1). Total RNA was extracted from ovaries of scallops at the growth stage using the RNAout column-based extraction kit (Sangon Biotech, Shanghai, China). The extracted RNA was reverse-transcribed into cDNA using a cDNA synthesis kit (TAKARA, Kyoto, Japan) following the manufacturer’s instructions. Using the synthesized cDNA as a template and primers E3/E4, a 207 bp fragment of the *cf*-*EST* cDNA corresponding to nucleotides’ positions 590–796 was amplified. The purified PCR product was used as a template for in vitro synthesis of double-stranded RNA (dsRNA) using the T7 RNAi Transcription Kit (Vazyme, Nanjing, China) following the manufacturer’s protocol. dsRNA preparation: The transcription reaction was performed in a total volume of 20 µL, containing 8 µL NTP Mix, 2 µL 10× Transcription Buffer, 2 µL T7 Enzyme Mix, 0.5 µg each of two DNA templates (1:1 ratio), and RNase-free H_2_O. The mixture was incubated at 37 °C for 2 h in a PCR thermal cycler. For transcripts longer than 800 bp, the reaction was terminated by heating at 72 °C for 10 min, followed by gradual cooling to allow for dsRNA annealing. Subsequently, the transcription product was treated with 1 µL DNase I and 2 µL RNase T1 (10 U/µL, diluted from 100 U/µL with RNase T1 Dilution Buffer) in a final volume of 40 µL at 37 °C for 30 min to remove template DNA and single-stranded RNA. The quality and concentration of the *EST*-dsRNA were assessed by agarose gel electrophoresis and spectrophotometry.

### 2.3. dsRNA Injection and Sampling

Acclimated healthy *C. farreri* were randomly divided into three groups: a blank control group, a negative control group, and an experimental group, with 20 individuals per group. Each group was placed in 200 L seawater to start the experiment. Each group was housed in a single tank; three groups were placed in three different tanks, and biological replicates represent individual scallops. According to the RNAi injection method described by Liu et al. [24], multiple-site injections were performed at the adductor muscle of *C. farreri* using a microsyringe. Each individual received a total of 50 μg of dsRNA administered at three injection sites, 16–17 ul at each injection point. For the negative control group, the injection regimen was exactly matched, including both the number of sites and the volume injected per site. The experimental group was injected with dsRNA (50 μg dsRNA dissolved in 100 μL PBS), while the negative control group was injected with 100 μL PBS. A second injection was administered one week later. During the experimental period, scallops were placed under the same culture conditions and daily management procedures described in Section 2.1. Sampling was conducted on day 12 after interference. Scallops were dissected to collect gonadal tissues, and the digestive tract was removed. The gonads were rapidly divided into several portions: one portion was fixed in Bouin’s solution for subsequent histological sectioning, and the remaining portions were snap-frozen in liquid nitrogen and stored at −80 °C for total RNA extraction and hormone analysis. RNA extraction and ELISA analysis were performed on samples obtained from the same individual and from the corresponding tissue regions.

### 2.4. Validation of RNAi Efficiency by qRT-PCR

The expression level of the *EST* gene after RNAi was analyzed using quantitative real-time PCR (qRT-PCR). Specific qRT-PCR primers for *C. farreri EST* (F3/R3; Table 1) and the internal reference gene *β*-*actin* (F1/R1; Table 1) were designed. Amplification was performed on a Light Cycler^®^ 96 real-time PCR system (Roche, Basel, Switzerland) according to the manufacturer’s instructions. The PCR cycling conditions were as follows: initial denaturation at 95 °C for 10 min, followed by 40 cycles of denaturation at 94 °C for 15 s, annealing at 60 °C for 1 min, and extension at 72 °C for 30 s. Three biological replicates (three independent scallops from each group) were analyzed for each tissue, with two technical replicates per sample. The relative expression levels of the target gene were calculated using the 2^−ΔΔCt^ method. Statistical significance was assessed using one-way analysis of variance (ANOVA), followed by the least significant difference (LSD) multiple comparison test, with *p* < 0.05 considered statistically significant. Prior to ANOVA, the assumptions of normality and homogeneity of variances were verified using the Shapiro–Wilk test and Levene’s test, respectively.

### 2.5. Detection of the Ovarian Development Marker Gene vtg to Assess Ovarian Development After RNAi

The vitellogenin gene (*vtg*), a recognized biomarker of ovarian development, was analyzed by qRT-PCR to assess its expression level. Specific primers for the *C. farreri vtg* (F2/R2; Table 1) were designed. The qRT-PCR program was conducted as described in Section 2.4.

### 2.6. Extraction and Detection of Estrogen in Gonadal Tissues After RNAi in C. farreri

Steroid hormones were extracted from *C. farreri* gonadal tissues using the dichloromethane liquid–liquid extraction method described by Guo et al. [25] The level of E2 in gonadal tissues was determined using an enzyme-linked immunosorbent assay (ELISA) kit (Cayman Chemical, Ann Arbor, MI, USA), following the manufacturer’s instructions. Estradiol quantification by ELISA: All samples were assayed in duplicate at a minimum of two dilutions. Briefly, 50 µL of estradiol standards or samples was added to the appropriate wells, followed by 50 µL of estradiol AChE tracer and 50 µL of estradiol antiserum (except for NSB, TA, and blank wells). NSB wells received 100 µL of ELISA buffer (1X), and B_0_ wells received 50 µL of ELISA buffer (1X). The plate was covered and incubated for 2 h at room temperature on an orbital shaker. After incubation, wells were emptied and rinsed five times with wash buffer (1X); subsequently, 200 µL of Ellman’s reagent was added to each well, and 5 µL of tracer was added to the TA wells. The plate was developed in the dark on an orbital shaker for 60 min. Absorbance was then read at 414 nm using a microplate reader. Estradiol concentrations were determined from the standard curve generated by four-parameter logistic regression. Each sample was analyzed with two technical replicates. Estrogen content was defined as the mass of estrogen per gram of wet tissue (pg/g). Statistical differences in estrogen levels among gonadal tissues from different experimental groups were analyzed using one-way ANOVA, with *p* < 0.05 considered statistically significant.

### 2.7. Prokaryotic Expression of the C. farreri EST Recombinant Protein

#### 2.7.1. Construction of the Recombinant Plasmid

Based on the *C. farreri EST* gene sequence (GenBank: PX515006), *EST* CDS amplification primers E1/E2 were designed (Table 1). Ovarian cDNA from *C. farreri* at the growth stage was used as the template for PCR amplification, and the PCR products were analyzed by agarose gel electrophoresis. The target fragments were ligated into a T-vector and transformed into *Escherichia coli* DH5α competent cells. Transformants were screened on ampicillin-containing medium, and positive colonies were identified by colony PCR using universal T7 and T7t primers. Positive clones were sequenced by Sangon Biotech (Shanghai, China) Co., Ltd. The correctly sequenced *EST* insert and the pET-28a(+) expression vector were both digested with the restriction endonucleases *EcoR* I and *Hind* III. The digested vector and *EST* fragment were recovered and purified, ligated using T4 DNA ligase, and subsequently transformed into *E. coli* BL21 competent cells. Positive transformants were selected on kanamycin-containing medium and confirmed by colony PCR.

#### 2.7.2. Expression and Purification of EST Recombinant Protein

*E. coli* cells harboring the recombinant plasmid were inoculated into LB medium containing kanamycin and cultured with shaking until the OD_600_ reached 0.6–0.8. Protein expression was induced by the addition of Isopropyl-beta-D-thiogalactopyranoside (IPTG) (Sangon Biotech, Shanghai, China) to a final concentration of 0.8 mmol/L, followed by incubation at 37 °C for 0, 2, 4, and 6 h. Bacterial cells were harvested by centrifugation, washed with PBS, and resuspended. The cells were disrupted by ultrasonication, and the pellet was collected. Inclusion bodies were solubilized using a urea denaturant. Protein expression was analyzed by SDS–PAGE. The target protein was purified by affinity chromatography using a Ni-NTA agarose resin purification kit (Sangon Biotech, Shanghai, China), and the purity of the eluted protein was evaluated by SDS–PAGE.

#### 2.7.3. Renaturation and Concentration of EST Recombinant Protein

Refolding of the protein was performed using the dilution refolding method, with reference to the composition of the basic refolding buffer described by Ma et al. [26]. Based on the physicochemical properties of the EST protein—specifically the isoelectric point, average hydrophilicity, and instability index analyzed using the ProtParam tool (ExPASy-ProtParam Tool. 2017. https://web.expasy.org/protparam/, accessed on 1 September 2024)—the refolding buffer conditions were adjusted to pH 8.0, supplemented with 5% glycerol, 1 M urea, and 0.2 M arginine. Following refolding, salts were removed by dialysis, and the protein solution was concentrated using ultrafiltration. Protein integrity was subsequently analyzed by SDS–PAGE.

### 2.8. Activity Assay of EST Recombinant Protein

The following experimental procedures have been filed for a Chinese patent (Patent application No.202610198378.X). Briefly, based on the reaction system of EST with E2 described by Nishiyama et al. [17], the final system was optimized to contain a dithiothreitol (DTT) (Beyotime, Shanghai, China) concentration of 0.05 mM and a dimethyl sulfoxide (DMSO) (Beyotime, Shanghai, China) content of 0.33%.Three experimental groups were set up: a blank control group (containing culture medium, cells, and PAPS), a negative control group (containing culture medium, cells, PAPS, and E2), and an experimental group (containing culture medium, cells, PAPS, E2, and EST). MCF-7 cells were cultured in phenol red-free DMEM (BasalMedia, Shanghai, China) supplemented with hormone-depleted (charcoal-stripped) serum for 1 week to eliminate the interference of endogenous estrogens. Cells in the logarithmic growth phase were harvested and seeded into 96-well plates. Following cell adherence, the culture medium was replaced with the respective treatment medium, and the cells were incubated for an additional 24 h. The effects of different reaction systems on cell proliferation were evaluated using the Cell Counting Kit-8 (CCK-8) assay (Beyotime, Shanghai, China), and the results were expressed as proliferation rate (PR%). The PR% was calculated as follows:

$$\mathrm{PR}\%=\frac{{\mathrm{OD}}_{\mathrm{experimental}}-{\mathrm{OD}}_{\mathrm{blank}\;\mathrm{control}}}{{\mathrm{OD}}_{\mathrm{negative}\;\mathrm{control}}-{\mathrm{OD}}_{\mathrm{blank}\;\mathrm{control}}}\times 100\%$$

### 2.9. Statistical Methods

All experiments were performed in three independent biological replicates. Statistical significance among the experimental, negative control, and blank control groups was evaluated using one-way analysis of variance (ANOVA), followed by least significant difference (LSD) post hoc testing for multiple comparisons. *p* < 0.05 was considered statistically significant. Prior to ANOVA, the assumptions of normality and homogeneity of variances were verified using the Shapiro–Wilk test and Levene’s test, respectively. Data processing and graphical visualization were carried out using Microsoft Excel. The above analyses were performed using PSPP software version 1.4.0.

## 3. Results

### 3.1. Assessment of RNAi Knockdown Efficiency in C. farreri

The Shapiro–Wilk and Levene’s tests confirmed that all data met the assumptions of normality and homogeneity of variance (*p* > 0.05 for all groups), permitting the use of parametric ANOVA.

**Figure 1:**
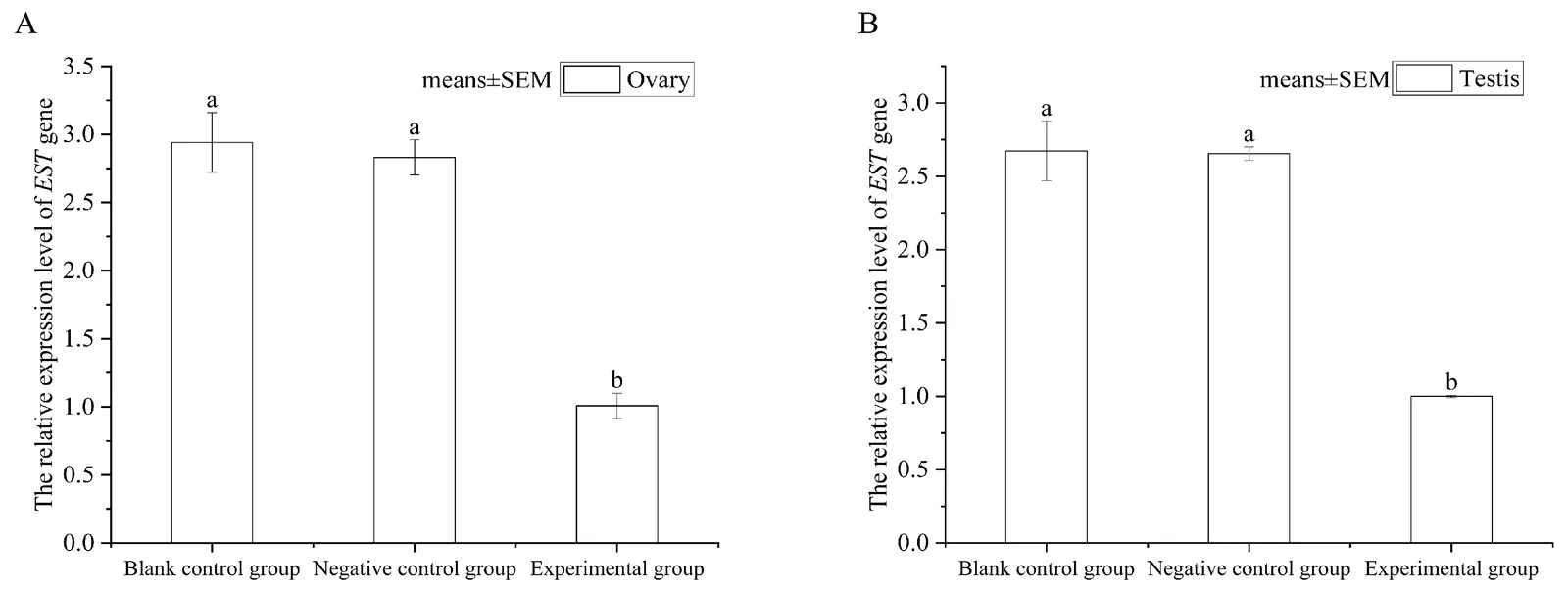
The level of *EST* mRNA detected by qRT-PCR in *C. farreri* ovaries (**A**) and testes (**B**) after RNAi. The expression level in the experimental group was set as 1.00; different letters (a and b) indicate statistically significant differences (*p* < 0.05).

qRT-PCR analysis showed that, compared with the blank control group, *EST* gene expression levels in the ovary and testis were significantly reduced by 67% ± 0.219 and 63% ± 0.204, respectively (*p* < 0.05). Similarly, relative to the negative control group, *EST* expression was significantly decreased by 65% in the ovary and 62% in the testis (Figure 1A,B), indicating successful knockdown of the *EST* gene (*p* < 0.05). No significant difference in *EST* expression was observed between the two control groups, suggesting that the injection procedure itself did not affect *EST* gene expression levels (*p* > 0.05).

### 3.2. Ovarian Development After RNAi Assessed by vtg Expression

**Figure 2:**
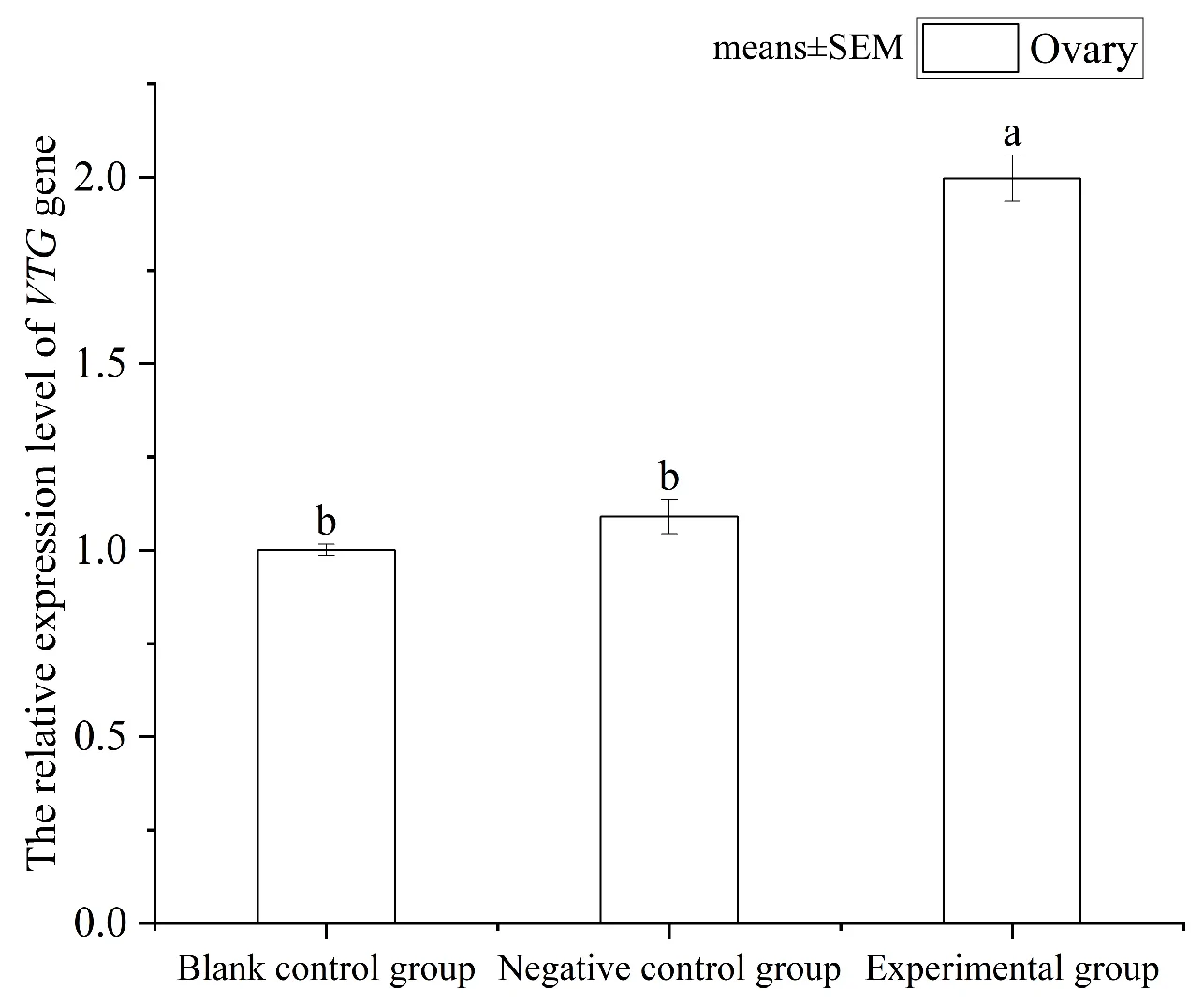
Relative expression of *vtg* mRNA in the ovaries of *C. farreri*. The expression level in the ovary of the blank control group was set as 1.00; different letters (a and b) indicate statistically significant differences (*p* < 0.05).

qRT-PCR analysis showed that *vtg* mRNA expression in the ovaries of the RNAi experimental group was significantly higher, 50% ± 0.015, than that in the blank control and 45.4% ± 0.046 higher than that in the negative control groups at the same time point (*p* < 0.05), whereas no significant difference was observed between the two control groups (Figure 2).

### 3.3. Effect of EST Gene RNAi on Gonadal Development Speed

#### 3.3.1. Effects of RNAi on EST Function

**Figure 3:**
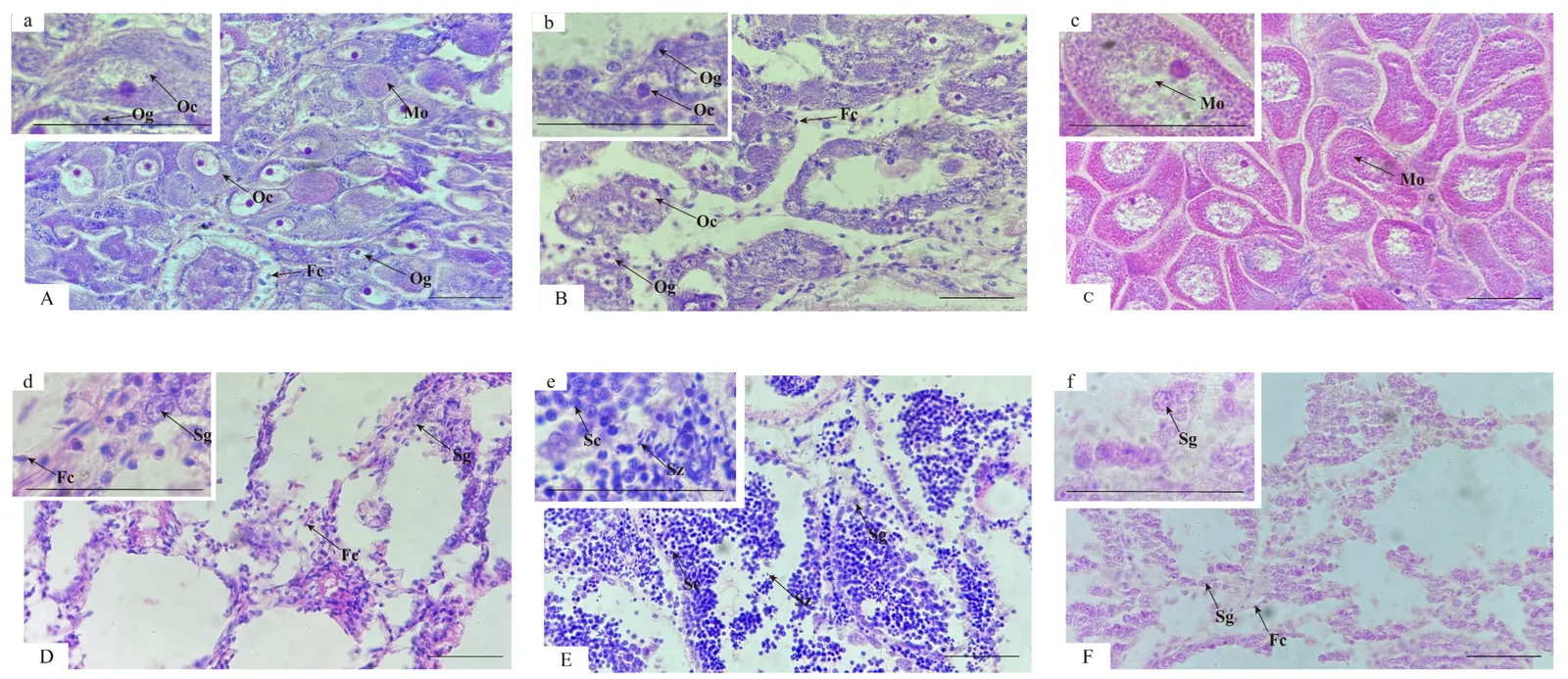
Histological observation by means of H&E staining method of *C. farreri* gonads effected by *EST* RNAi. (**A**) Ovary (experimental group); (**B**) ovary (control group); (**C**) ovary (normal mature stage); (**D**) testes (experimental group); (**E**) testes (control group); (**F**) testes (normal early growth phase); Og, oogonia; Oc, oocyte; Mo, mature oocyte; Sg, spermatogonia; Sc, spermatocyte; Sz, spermatozoon; Fc, follicular cell; (**a**–**f**) show magneified views of (**A**–**F**); scale bar = 50 μm.

Histological examination of the gonads after RNAi showed that, in the experimental group, the ovarian follicles were entirely filled with germ cells, with no visible lumen, and the interfollicular spaces were largely absent (Figure 3A–C). The mean diameter of oocytes in the experimental group was 32.11 ± 4.6 µm (*n* = 20), while that of the control group was 13.9 ± 1.4 µm (*n* = 20), and that of the mature oocytes was 50.3 ± 7.4 µm (*n* = 20). Compared with the control group, oocytes in the experimental group exhibited a larger diameter, and a subset of oocytes displayed morphological characteristics similar to mature oocytes (first, the entire follicular lumen is filled with germ cells, leaving few visible empty spaces within the follicles; second, mature oocytes are packed tightly and compress each other, resulting in irregular shapes such as elliptical, pyriform, or polygonal forms; third, the oocytes’ diameter is also larger than that at the growth period), including an elongated nucleus and condensed cytoplasm. As shown in Figure 3D–F, spermatocytes had just begun to appear within the testicular follicles of the experimental group, and the follicular lumen remained largely empty. In contrast, the follicular lumen of the control group was progressively filled with germ cells, and spermatozoa were observed. In the control group, germ cells at multiple developmental stages, ranging from spermatogonia to spermatozoa, were present within the follicles, and the arrangement of germ cells transitioned from a single-layered to a multilayered organization.

#### 3.3.2. Estrogen Content in Gonadal Tissue Following RNAi Treatment

**Figure 4:**
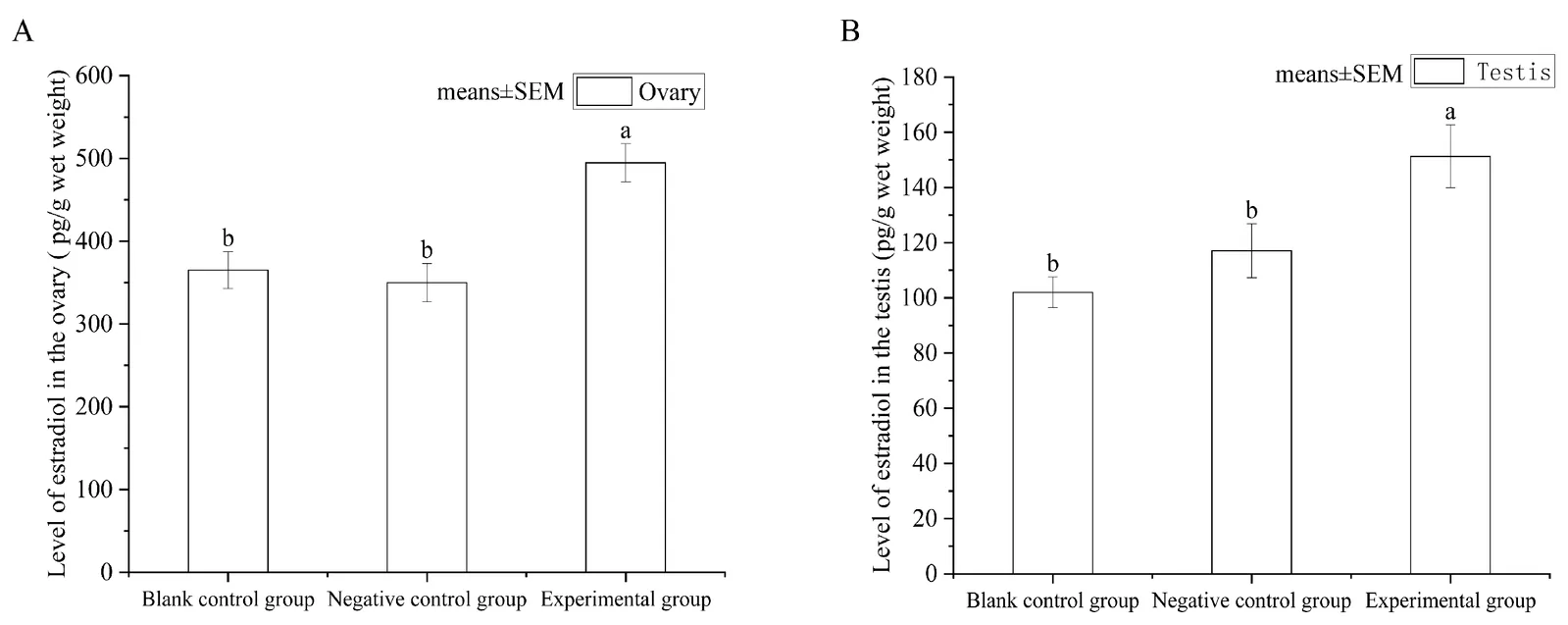
(**A**) Estradiol content in the ovary of *C. farreri* after RNAi treatment. (**B**) Estradiol content in the testis of *C. farreri* after RNAi treatment. Different letters (a and b) indicate statistically significant differences (*p* < 0.05).

In ovaries, E2 levels in the experimental group were significantly higher than in the blank control and negative control groups (*p* < 0.05), whereas no significant difference was observed between the two control groups (Figure 4A). For the ovary, the E2 content in the experimental group was 1.414-fold that of the negative control group and 1.356-fold that of the blank control group. A similar pattern was observed in testes, where E2 levels in the experimental group were significantly higher than those in both control groups, with no significant difference between the control groups (Figure 4B). For the testis, the E2 content in the experimental group was 1.292-fold that of the negative control group and 1.483-fold that of the blank control group. These results are consistent with the histological observations described above.

### 3.4. Prokaryotic Expression Results of EST Recombinant Proteins from C. farreri

#### 3.4.1. Expression of Recombinant EST Protein Following Induction

**Figure 5:**
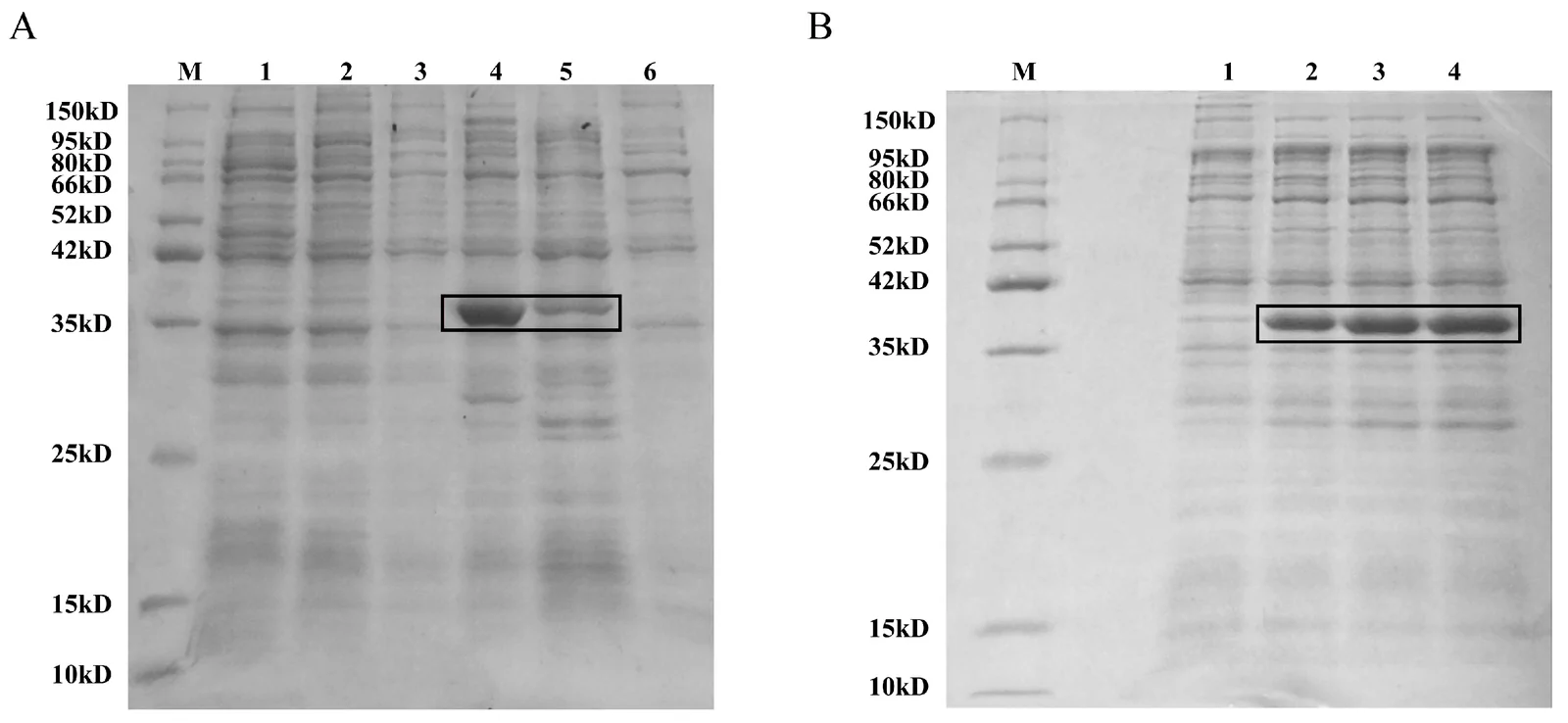
SDS-PAGE detection of EST recombinant protein induced expression. (**A**) Expression forms of recombinant proteins. M: protein marker; 1: empty plasmid whole bacterial solution; 2: recombinant plasmid did not induce whole bacterial solution; 3: recombinant plasmid induced whole bacterial solution at 0 h; 4: recombinant plasmid whole bacterial solution; 5: recombinant plasmid bacterial solution is precipitated after crushing; 6: supernatant after crushing recombinant plasmid bacterial solution. (**B**) Time optimization for recombinant protein induction expression. M: protein marker; 1–4: recombinant plasmid induced whole bacterial solution at 0 h, 2 h, 4 h, and 6 h. The black box is the target protein.

Following IPTG induction of the recombinant plasmid pET-28a-*EST*, SDS-PAGE analysis revealed a distinct protein band at approximately 35 kDa in the experimental group (Figure 5A), consistent with the predicted molecular weight of 35.3 kDa. This result indicated successful expression of the *EST* gene in *E. coli* BL21 competent cells, with the recombinant EST protein predominantly present in the form of inclusion bodies. Analysis of products obtained at different induction times showed that EST protein expression reached its maximum level after 4 h of IPTG induction (Figure 5B).

#### 3.4.2. Purification Results of the Recombinant EST Protein

**Figure 6:**
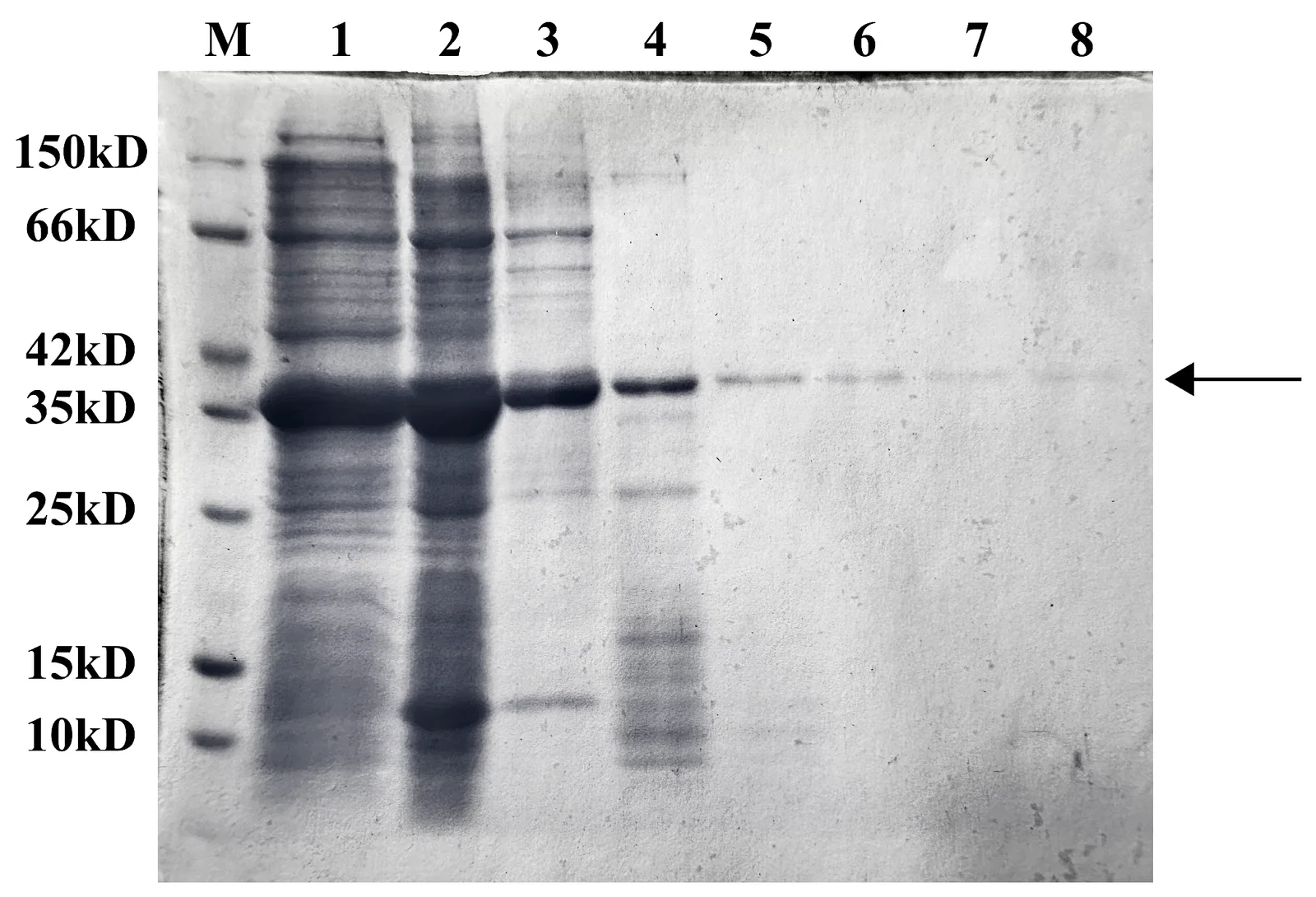
SDS-PAGE detection of recombinant protein purification. M: protein marker; 1: recombinant plasmid whole bacterial solution; 2: inclusion body solution; 3: effluent after protein upper column; 4–8: EST protein eluent purified by nickel column. The arrow indicates the target protein.

Following purification of the recombinant EST protein using a Ni-NTA column, SDS-PAGE analysis of the eluted fraction revealed a single protein band at approximately 35 kDa (Figure 6), indicating successful purification of the EST protein.

#### 3.4.3. Refolding and Concentration Results of the Recombinant EST Protein

**Figure 7:**
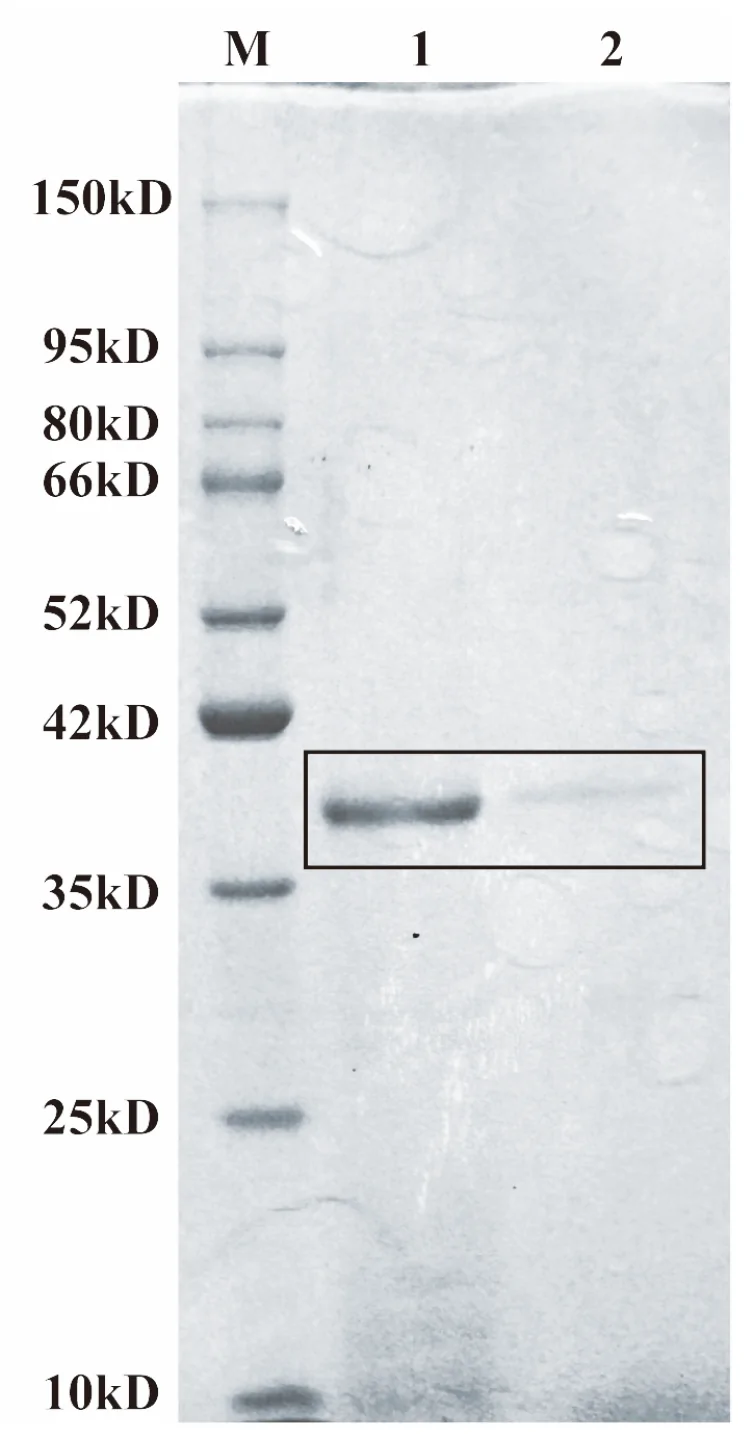
SDS-PAGE detection of recombinant EST protein by dilution method. M: protein marker; 1: 8M urea dissolved denatured protein solution; 2: dilute the concentrated protein supernatant after renaturation. The black box is the target protein.

After refolding, the recombinant EST protein was detected as a band corresponding to the expected molecular weight in the concentrated protein supernatant, indicating successful refolding (Figure 7).

### 3.5. Activity Assay of the Recombinant EST Protein

**Figure 8:**
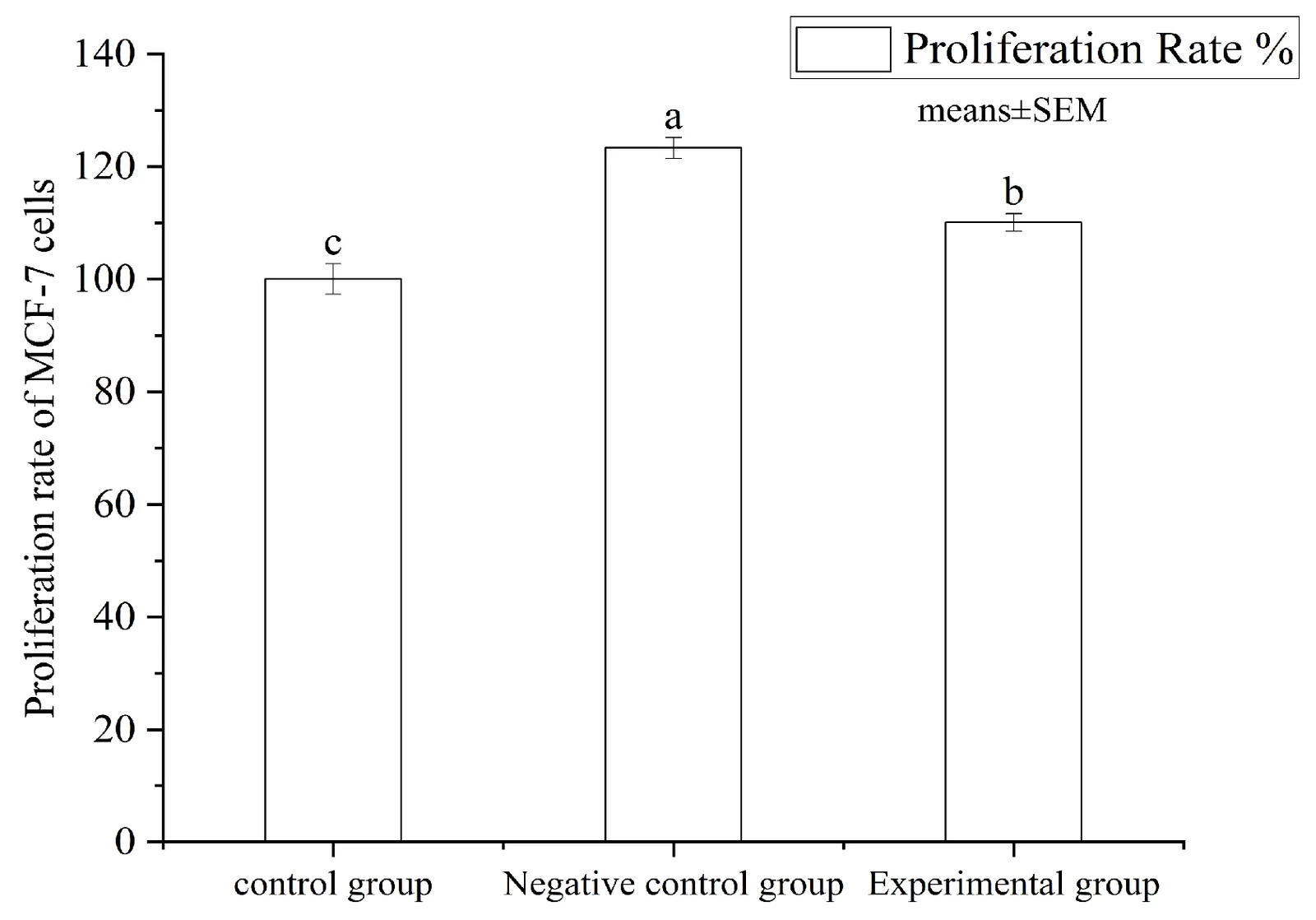
Histogram of MCF-7 cell proliferation rate in different treatment groups. Different letters indicate significant differences (*p* < 0.05).

Among the three groups, MCF-7 cell proliferation rates were highest in the negative control group, followed by the experimental group, and lowest in the blank control group, with significant differences observed between groups (*p* < 0.05), indicating that the recombinant EST protein was active (Figure 8).

## 4. Discussion

At present, research on *EST* has been mainly focused on vertebrates, while studies in invertebrates remain scarce. In this study, *EST* from *C. farreri* was investigated. Previous work showed that the conserved domains of *cf*-*EST* are similar to vertebrate *EST*, including the dimerization domain, PAPS-binding region, and conserved residues (Ser, Lys, His). However, *cf*-*EST* differs from vertebrate *EST* in the substrate-binding pocket “gate” structure. In vertebrates, the conserved amino acids at this site are typically a preceding Tyr and a following Phe (or both Phe) [27,28], whereas in *cf*-*EST*, the corresponding residues are a preceding Phe and a following Tyr. Moreover, our earlier qRT-PCR analyses showed that *cf*-*EST* is predominantly expressed in the gonads, suggesting that its primary function is localized to this tissue [20]. It is well established that the proper function of proteins generally relies on the integrity of conserved regions within their amino acid sequences. Whether the sequence differences observed in the conserved domain of *cf*-*EST* affect its enzymatic function or regulatory mechanisms remains to be elucidated. This question forms the basis of the present study.

In vertebrates, *EST* plays a critical role in reproductive regulation, as evidenced by the finding of Gershon et al. [14] that genetic ablation of *EST* in female mice led to ovulatory defects and a marked reduction in fertility. This effect was attributed to elevated estrogen levels after *EST* loss. Correspondingly, following *cf*-*EST* knockdown, histological observations revealed that the number of oocytes in the ovaries of the experimental group was significantly higher than that in the control group (*p* < 0.05), with some oocytes exhibiting diameters approaching those of mature oocytes. Compared with the blank control group, the EST gene expression levels in the ovary and testis were significantly reduced by 67% ± 0.219 and 63% ± 0.204, respectively (*p* < 0.05).According to the description of the ovarian growth stage in *C. farreri* by Liao et al. [22], these morphological characteristics indicate that ovarian development in the experimental group proceeded at a faster rate than in the control group. Previous studies have suggested that, in higher mammals, *EST* mainly exerts its biological functions by regulating the level and activity of E2. Wang and Croll [29] reported that injection of E2 in *Placopecten magellanicus* resulted in the appearance of abnormally enlarged oocytes in the ovary, which were morphologically similar to mature oocytes and characterized by nuclear elongation and cytoplasmic condensation; the authors concluded that exogenous E2 could promote ovarian development. In addition, Liu et al. [30] quantified E2 levels in *C. farreri* ovaries at different stages throughout the year using ELISA and compared these values with oocyte diameters, demonstrating that E2 stimulates oocyte growth and that ovarian E2 content is positively correlated with oocyte diameter. Collectively, these studies consistently indicate that E2 plays a crucial role in promoting oocyte growth and ovarian development. The results of the present study are highly consistent with these findings, showing that EST-dsRNA treatment increased ovarian E2 content 1.414-fold relative to the negative control and 1.356-fold relative to the blank control, suggesting that *EST*-dsRNA treatment may enhance ovarian development by increasing the biological activity of E2 within the ovary. In testes, histological analysis showed that the control group had testes at mid-development, while experimental testes lagged in early development, with most germ cells remaining spermatogonia [22]. Similar phenomena have been reported in vertebrates, as Li et al. [31] suggested that high-dose E2 injection in cryptorchid mice promoted spermatogonial division, indicating a dose-dependent mitogenic effect. Other studies showed that E2 reduces apoptosis and stimulates proliferation of human spermatogonia in vitro [32], and promotes spermatogonial proliferation in vivo in rats [29,33]. Based on these findings, we propose that *EST*-dsRNA treatment may elevate E2 levels in testes, stimulating spermatogonia division while potentially delaying differentiation into spermatocytes. In vertebrate testes, estrogens can also suppress androgen production via paracrine–autocrine mechanisms [34]. For the testis, the E2 content in the experimental group was 1.292-fold that of the negative control group and 1.483-fold that of the blank control group. By analogy, *cf*-*EST* knockdown may increase estrogen activity in *C. farreri*, inhibiting normal testis development and preventing progression from early to mid-development. Overall, these results indicate that *cf*-*EST* mainly functions to inactivate estrogen activity in gonads. Measurement of gonadal estrogen after RNAi treatment further showed that *cf*-*EST* reduces overall estrogen levels. Thus, *cf*-*EST* regulates gonadal development by modulating both estrogen activity and content, a function highly consistent with vertebrate *EST*. We acknowledge that sulfated estrogen metabolites were not directly measured in this study. Therefore, our mechanistic interpretation relies on inferred EST activity and should be considered preliminary until confirmed by direct metabolite quantification in future work.

Previous studies have shown that in higher mammals, *EST* functions mainly by catalyzing the sulfation of estrogens, thereby rendering them biologically inactive. Schuler [35] proposed that, in humans, sulfated steroids generally follow two metabolic fates: one involves elimination from the body through excretory pathways, while the other entails entry into the circulation in the form of steroid sulfates, which serve as precursor reservoirs transported to specific target organs and provide essential substrates for efficient local steroid synthesis. Both pathways reduce active steroid levels in the tissue. In this study, *cf*-*EST* knockdown resulted in elevated estrogen levels in the gonads, consistent with the mammalian mechanism, thereby suggesting that *cf*-*EST* regulates gonadal development by modulating E2 content. It also indicates that differences in the substrate-binding pocket “gate” residues between *cf*-*EST* and vertebrate *EST* do not impair *cf*-*EST* function.

Beyond histological analysis, gonadal development is commonly evaluated using molecular markers, among which the *vtg* gene has been established as a critical indicator of ovarian development in *C. farreri*.

Xing et al. [36] used *vtg* to monitor ovarian maturation in *C. farreri*. Qin et al. [37] analyzed *cf*-*vtg* expression during the ovarian cycle of *C. farreri* using qRT-PCR and assessed its response to E2 injection, showing that *cf*-*vtg* expression was highest at maturation, intermediate in growth, and lowest in proliferation, and was significantly upregulated by E2, indicating that *cf*-*vtg* expression reflects oogenesis and is inducible by E2. In this study, *cf*-*vtg* was used as a molecular marker to complement histological observations and assess ovarian development after RNAi treatment. Although molecular markers for *C. farreri* testis are limited, changes in ovarian *cf*-*vtg* expression can reflect the overall effect of RNAi on gonadal development. Furthermore, *EST* knockout in mice has been reported to cause structural and functional abnormalities in the male reproductive system [38], which is consistent with the delayed testis development observed in the present study and further supports an important regulatory role of *cf*-*EST* in testis development.

Most studies on *EST* mechanisms have focused on humans. Rubin et al. [16] expressed human EST in a prokaryotic system and used isotope labeling to validate its mechanism. Nishiyama et al. [17] similarly studied recombinant human EST and its effect on estrogen activity using isotope labeling. These studies demonstrate that human EST catalyzes 3′-hydroxyl sulfation of estrogen using PAPS as the sulfate donor, thereby inactivating the hormone, and that mechanistic investigations of EST have largely relied on recombinant proteins. In this study, *cf*-*EST* was successfully expressed in *E. coli*, providing a foundation for functional analysis. Based on estrogen as an EST substrate, estrogen activity can be measured using the E-SCREEN assay [39], which evaluates proliferation of estrogen-sensitive cells such as MCF-7. For example, Wang et al. [40] showed that *Paulownia tomentosa* flower extracts (1–10 μg/mL) promoted MCF-7 proliferation, indicating estrogenic activity. Tatay et al. [41] demonstrated that zearalenone and derivatives significantly induced MCF-7 proliferation, further validating the method. We incubated recombinant *cf*-*EST* with estrogen and the sulfate donor PAPS in vitro, and then measured estrogen activity using E-SCREEN. EST treatment significantly reduced estrogen-induced proliferation, confirming that *cf*-*EST* can inactivate estrogen enzymatically. These results suggest that *cf*-*EST* may share a mechanism with human EST, using PAPS to catalyze estrogen sulfation and reduce its activity.

Traditional EST activity assays often rely on isotope labeling [16,17,18], which requires careful consideration of cost, safety, feasibility, and data reliability. In this study, we developed a simpler, cost-effective, non-radioactive method based on estrogen as the EST substrate. This approach may also facilitate EST activity analysis in other mollusks and provide a foundation for investigating *EST* genes across diverse species.

## 5. Conclusions

In this study, RNAi experiments demonstrated that the *EST* gene of *C. farreri* plays an important regulatory role in gonadal development, and that its normal expression is required for proper gonadal progression. Recombinant EST protein was produced using a prokaryotic expression system, and following refolding and concentration, its enzymatic activity was verified using a newly established activity assay. Using ELISA to quantify gonadal E2 levels following RNAi treatment, the results indicate that *C. farreri EST* regulates both the activity and content of E2 and, through this regulation, indirectly influences gonadal development. Although the conserved amino acid residues in the substrate-binding “gate” region of *C. farreri EST* differ from those of vertebrate *EST*, these differences do not affect enzymatic function or alter the protein’s mode of action, indicating that *C. farreri EST* is functionally and mechanistically similar to its homologs in higher mammals.

Indeed, numerous proteins play roles in invertebrate reproductive biology, and a better understanding of these proteins will lay the foundation for mechanistic studies on invertebrate reproduction. This is particularly true for commercially important marine aquaculture species, where research on these proteins will not only contribute to basic theoretical studies on reproductive and developmental mechanisms, but also provide practical guidance for seedling production and hatchery rearing, thereby holding significant research value. In the present study, our findings on EST indicate that it is involved in hormonal regulation in the scallop and affects gonadal development, which may provide a basis for the artificial regulation of reproductive timing. In addition, the novel technique developed in this study offers a new approach for detecting EST enzymatic activity. For example, our team has applied this technique to commercially available recombinant human EST and confirmed its feasibility and reliability.

## Figures and Tables

**Table 1:** Primer sequence.

Gene	Primer	Primer Sequence (5′→3′)	Melting Temperature	Usage
*EST*	E1	CCGGAATTCATGCCGTACAAAATT	54	PCR
	E2	CCCAAGCTTTTACAGCAGACAGGTG	60.2	
	E3	TAATACGACTCACTATAGGGATCCCATACATGTTGTCAAT	64.3	RNAi
	E4	TAATACGACTCACTATAGGGTAGGGTACTTGTTATCTAGAAAGT	64.8	
	F3	CGTGTGATGAAGGTCAAGGG	54.4	qPCR
	R3	TACACATCGCCACAGAAGCA	52.4	
*vtg*	F1	TTCTTGGGAATGGAATCTGC	52.4	qPCR
	R1	GCCAGACTCGTCGTATTCCT	52.4	
*β-actin*	F2	GCCCAATCAAAAGAGAGCTG	58.89	qPCR
	R2	GTCTGCAGCATCTGGACAAA	58.12	

Note: Bases indicated with double underlines represent protective nucleotides; bases with single underlines correspond to the recognition sites for the restriction enzymes *EcoR I* and *Hind III*. The T7 promoter sequence is indicated with a wavy underline.

## Data Availability

In the next few years, several graduate students in our laboratory will conduct further research based on these omics data. If the total data are leaked in advance, it will affect our laboratory follow-up research. So, based on the above considerations and the policies and confidentiality agreements adhered to in our laboratory, we cannot provide the total raw data. If anyone has questions about specific data, they can contact the corresponding author, and we will do our best to provide more detailed elaboration.
